# Angiogenic and Osteogenic Synergy of Human Mesenchymal Stem Cells and Human Umbilical Vein Endothelial Cells Cocultured on a Nanomatrix

**DOI:** 10.1038/s41598-018-34033-2

**Published:** 2018-10-24

**Authors:** Jun Chen, Lily Deng, Catherine Porter, Grant Alexander, Dhruv Patel, Jeremy Vines, Xixi Zhang, David Chasteen-Boyd, Hak-Joon Sung, Yi-Ping Li, Amjad Javed, Shawn Gilbert, Kyounga Cheon, Ho-Wook Jun

**Affiliations:** 1 Department of Biomedical Engineering, University of Alabama at Birmingham, Birmingham, Alabama 35294 United States; 2 Department of Medical Engineering, Severance Biomedical Science Institute, College of Medicine, Yonsei University, Seoul, 120-752 Korea; 3 Department of Pathology, University of Alabama at Birmingham, Birmingham, Alabama 35294 United States; 4 Department of Oral and Maxillofacial Surgery, University of Alabama at Birmingham, Birmingham, Alabama 35294 United States; 5 Department of Orthopedic Surgery, University of Alabama at Birmingham, Birmingham, Alabama 35294 United States; 6 Department of Pediatric Dentistry, University of Alabama at Birmingham, Birmingham, Alabama 35294 United States

## Abstract

To date, bone tissue regeneration strategies lack an approach that effectively provides an osteogenic and angiogenic environment conducive to bone growth. In the current study, we evaluated the osteogenic and angiogenic response of human mesenchymal stem cells (hMSCs) and green fluorescent protein-expressing human umbilical vein endothelial cells (GFP-HUVECs) cocultured on a self-assembled, peptide amphiphile nanomatrix functionalized with the cell adhesive ligand RGDS (PA-RGDS). Analysis of alkaline phosphatase activity, von Kossa staining, Alizarin Red quantification, and osteogenic gene expression, indicates a significant synergistic effect between the PA-RGDS nanomatrix and coculture that promoted hMSC osteogenesis. In addition, coculturing on PA-RGDS resulted in enhanced HUVEC network formation and upregulated vascular endothelial growth factor gene and protein expression. Though PA-RGDS and coculturing hMSCs with HUVECs were each previously reported to individually enhance hMSC osteogenesis, this study is the first to demonstrate a synergistic promotion of HUVEC angiogenesis and hMSC osteogenesis by integrating coculturing with the PA-RGDS nanomatrix. We believe that using the combination of hMSC/HUVEC coculture and PA-RGDS substrate is an efficient method for promoting osteogenesis and angiogenesis, which has immense potential as an efficacious, engineered platform for bone tissue regeneration.

## Introduction

Bones not only provide support, but they also regulate blood pH, act as a mineral reservoir, generate hematopoietic stem cells, and produce mesenchymal stem cells^1–3^. Each year, delayed union and nonunion inhibit the healing process of 5–10% of the approximately 8 million incidences of bone fracture in the U.S. alone^4^. Due to the high importance of bone, finding strategies to aid in bone regeneration is vital. Currently, bone grafts are used as a standard clinical treatment for bone defects^5^. However, avascular bone grafts depend on diffusion for nutrient supply; therefore, large bone grafts often receive inadequate nutrition via diffusion, which leads to cell death^5^. Furthermore, resorption of the graft frequently occurs faster than osteogenesis. Autografts, in particular, are associated with donor site morbidity, and allografts increase the risk of introducing infection or disease^6^. To overcome the inherent problems with grafts, an alternative approach to assist in the healing of critical-size bone defects is to utilize a construct that mimics the natural bone microenvironment, which consists of inorganic hydroxyapatite crystals, organic protein fibers, osteogenic cells, and angiogenic cells^7,8^.

A bone analogous scaffold should contain components that not only promote osteogenesis but also foster angiogenesis to prevent hypoxia-induced cell death^9^. In bone tissue engineering, human mesenchymal stem cells (hMSCs) are commonly used as osteoprogenitor cells that can differentiate into osteoblasts and regenerate bone, and endothelial cells (ECs), often from umbilical veins, are used for angiogenesis. A main advantage of using hMSCs is that their endogenous production of angiogenic cytokines eliminates the need for the exogenous administration of therapeutic soluble factors that can induce angiogenesis in untargeted tissues, stimulate neoplastic growth, promote the development of abnormally functioning blood vessels, and increase atherosclerotic plaque mass^10^.

Because osteoprogenitor cells and ECs both play vital roles in bone regeneration, many studies have investigated the effects of communication between these two cells on osteogenesis and angiogenesis^11–21^. For instance, it has been reported that in cocultures of hMSCs and ECs, direct cell-cell interactions and the paracrine effects induced by EC cytokines and regulatory molecules can enhance hMSC osteogenic differentiation^15–17,22,23^. Additionally, the Unger group showed that coculturing hMSC-derived osteoblasts with dermal microvascular ECs forms tissue-like structures with microcapillary-like networks^18^. Furthermore, Ma *et al*. demonstrated that the highest levels of osteogenic differentiation and angiogenesis are obtained in cocultures of hMSCs and human umbilical vein endothelial cells (HUVECs) at a cell ratio of 50:50 in differentiation medium^17^. Lastly, it was reported that hMSCs can stabilize EC-formed vascular structures *in vivo* and *in vitro*, inhibit EC apoptosis, and stimulate angiogenesis^19,20^.

Due to the mutual enhancements in osteogenesis and angiogenesis observed in hMSC/EC cocultures, investigating the effects of scaffold properties on osteogenesis and angiogenesis in cocultures has attracted much attention^12^. Notably, Kim *et al*. showed that coculturing hMSCs with HUVECs on nanotopography enhances hMSC osteogenesis more than culturing hMSCs alone on nanotopographical substrates or in coculture with ECs on flat substrates^15^. Stoppato *et al*. reported that enhanced osteogenic differentiation was observed in hMSC/HUVEC cocultures on silk fibroin-free scaffolds compared with hMSCs cultured alone or on scaffolds with silk fibroin; in contrast, coculturing on silk fibroin-coatings promoted EC growth^14^. Furthermore, Guerrero’s group demonstrated that the combination of hMSC/EC coculture and 3D microenvironment—provided by a polysaccharide-based scaffold—favored hMSC osteogenesis^21^. In summary, each of these described tailored scaffolds has had substantial effects in stimulating either hMSC osteogenesis or HUVEC angiogenesis in cocultures.

Inspired by previous studies, in the current study, we introduced green fluorescent protein-expressing HUVECs (GFP-HUVECs), an angiogenic component, into hMSC cultures on a peptide amphiphile (PA-RGDS) nanomatrix. This addition of HUVECs helped us to develop an *in vitro* environment that more closely recapitulates conditions which would be found in the future *in vivo* studies. In such *in vivo* studies, PA-RGDS, ECs, and hMSCs are expected to directly interact with one another. We investigated (1) the synergistic effects of the PA-RGDS nanomatrix and coculture with HUVECs on hMSC osteogenesis, and (2) the synergistic effects of the PA-RGDS nanomatrix and coculture with hMSCs on HUVEC angiogenesis.

As described in preceding literature, PA-RGDS nanomatrix contains a hydrophobic alkyl chain that is covalently linked to two hydrophilic sequences: (1) the matrix metalloproteinase-2 (MMP-2) gene sequence, which promotes cell-driven scaffold degradation and fosters cell migration; and (2) the Arg-Gly-Asp-Ser (RGDS) sequence, a cell adhesion ligand, found naturally in fibronectin, through which the nanofibers mediate additional cell-extracellular matrix and cell-cell interactions^24–26^. Moreover, due to its amphiphilic nature, PA-RGDS can self-assemble into highly organized cylindrical nanofibers. At a higher order level, PA-RGDS nanofibers intertwine to form a nanomatrix, which mimics the organic structural component of the extracellular matrix (ECM)^27–30^. Previously, we showed that in both growth and differentiation media, the PA-RGDS nanomatrix can increase osteogenic differentiation of hMSCs into osteoblasts^28–30^. Incorporating hydroxyapatite nanoparticles into the PA-RGDS nanomatrix can yet further promote hMSC osteogenic differentiation^30^.

**Figure 1:**
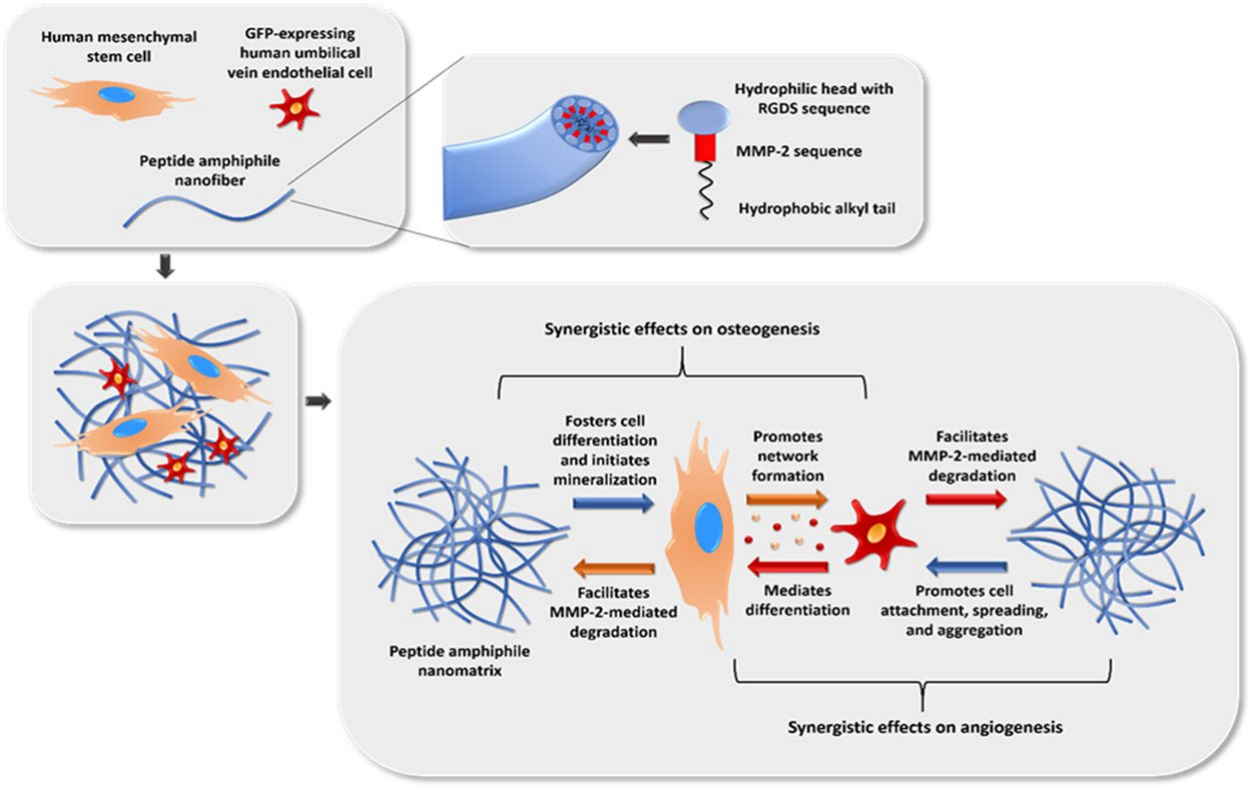
Schematic of the experimental strategy. Included are the expected synergistic effects on osteogenesis and angiogenesis of hMSCs and GFP-HUVECs cocultured on a peptide amphiphile nanomatrix functionalized with the RGDS motif.

Therefore, here, we expected that cocultures on PA-RGDS nanomatrix would synergistically promote osteoblastic differentiation and HUVEC angiogenesis. More specifically, we hypothesized that: (1) the coculture with hMSCs and the PA-RGDS nanomatrix would amplify the angiogenic response of HUVECs compared to their monoculture or coculture on the standard negative control, plasma-treated tissue culture plates (TCPs); and (2) hMSCs in coculture with HUVECs on PA-RGDS would show a greater osteogenic response than any other experimental group (Fig. 1). To address our hypotheses, PA-RGDS nanomatrix substrates were prepared first. Then, hMSC/GFP-HUVEC cocultures were maintained on PA-RGDS. The hMSC monocultures and GFP-HUVEC monocultures on PA-RGDS nanomatrix as well as the hMSC monocultures, GFP-HUVEC monocultures and hMSC/GFP-HUVEC cocultures on TCP were prepared as controls for comparison. Osteogenesis of hMSCs was evaluated by analyzing gene and protein expression as well as staining and quantifying calcium deposition. Angiogenesis by GFP-HUVECs was investigated by analyzing the gene and protein expression and by imaging GFP-HUVEC network formation. To the best of our knowledge, this is the first study that demonstrated the synergistic effects of cell-cell and cell-PA-RGDS interactions on hMSC osteogenesis and HUVEC angiogenesis.

## Results and Discussion

### Alkaline phosphatase assay

ALP is considered an early marker of osteoblastic differentiation^31,32^. It is widely used to detect osteoblasts because an increase in ALP activity can signify the start of new bone formation by these cells. During the differentiation process, ALP activity within cell cultures will increase when organic phosphates are cleaved to create free, inorganic phosphates for use in mineralization^33^. Enhanced expression of ALP occurs during ECM maturation, which precedes mineralization^34,35^.

**Figure 2:**
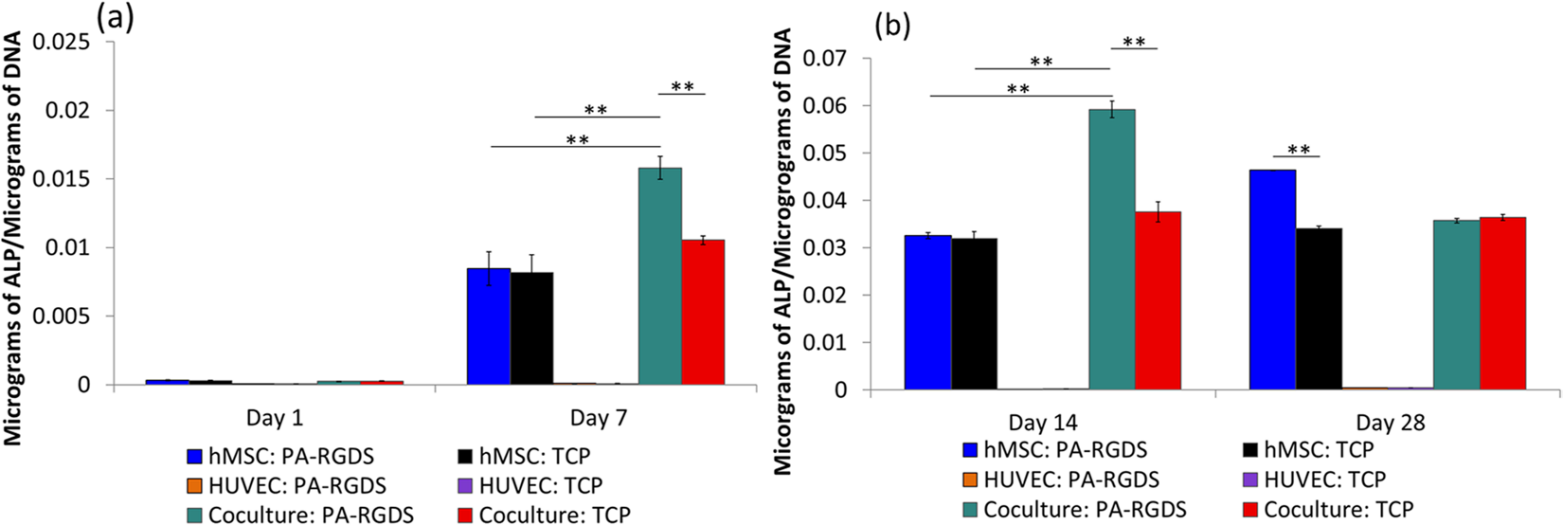
ALP activity at days 1 and 7 (**a**) and days 14 and 28 (**b**) post cell-seeding. Samples were normalized by total DNA content observed in the Picogreen assay. Values are expressed as a mean ± standard error of measurement (**p = 0.01).

Figure 2 shows the ALP activity measured in cultures at days 1, 7, 14, and 28. Because ALP is produced by osteogenic cells, observed ALP activity in cocultures is attributed to hMSCs and not HUVECs. Importantly, at days 7 and 14, hMSCs in cocultures generally showed slightly greater ALP activity compared to their monocultured counterparts. The respective ALP levels from hMSCs cocultured and monocultured on TCPs at day 7 were 0.011 ± 0.0006 and 0.008 ± 0.002 μg of ALP per μg of DNA. The ALP from hMSCs cocultured and monocultured on TCPs then increased at day 14 to 0.038 ± 0.005 and 0.032 ± 0.003 μg of ALP per μg of DNA, respectively. The enhanced osteogenesis that resulted from the coculture condition is in agreement with previous studies^22,36,37^. For instance, Hasirci’s group showed that cocultures of rat bone marrow stem cells and rat aortic endothelial cells exhibited more ALP activity than either cell in monoculture^22^. The addition of PA-RGDS yet further amplified ALP activity; cocultured hMSCs on PA-RGDS exhibited ALP activity at day 7 (0.016 ± 0.002 μg ALP per μg of DNA) and day 14 (0.059 ± 0.004 μg of ALP per μg of DNA) that was significantly greater than the ALP activity of any other condition at the same timepoints. Interestingly, the ALP activity for cocultured hMSCs on PA-RGDS progressively increased from day 1 to day 14 but significantly decreased by day 28. A similar result was also demonstrated by Ma *et al*., in which the highest ALP activity in hMSC/HUVEC cocultures occurred on day 14 with a cell ratio of 50:50^17^. However, the ALP activity for monocultured hMSCs continuously increased throughout the incubation period, and the highest ALP activity (0.046 ± 0.00015 μg of ALP per μg of DNA) was observed at day 28. A promising result, the earlier and greater ALP activity in the PA-RGDS cocultures signifies not only increased osteogenesis, but a more rapid overall differentiation process^38,39^. In addition, at day 28, the ALP activity of hMSC monocultures on PA-RGDS was higher than that of hMSC monocultures on TCPs which also agrees with our previous reported data^28^. Overall, the data indicate that PA-RGDS combined with the coculture condition synergistically promotes greater osteogenic differentiation of hMSCs than other conditions in the current study.

### Mineralization analysis via Alizarin Red quantification and von Kossa staining

**Figure 3:**
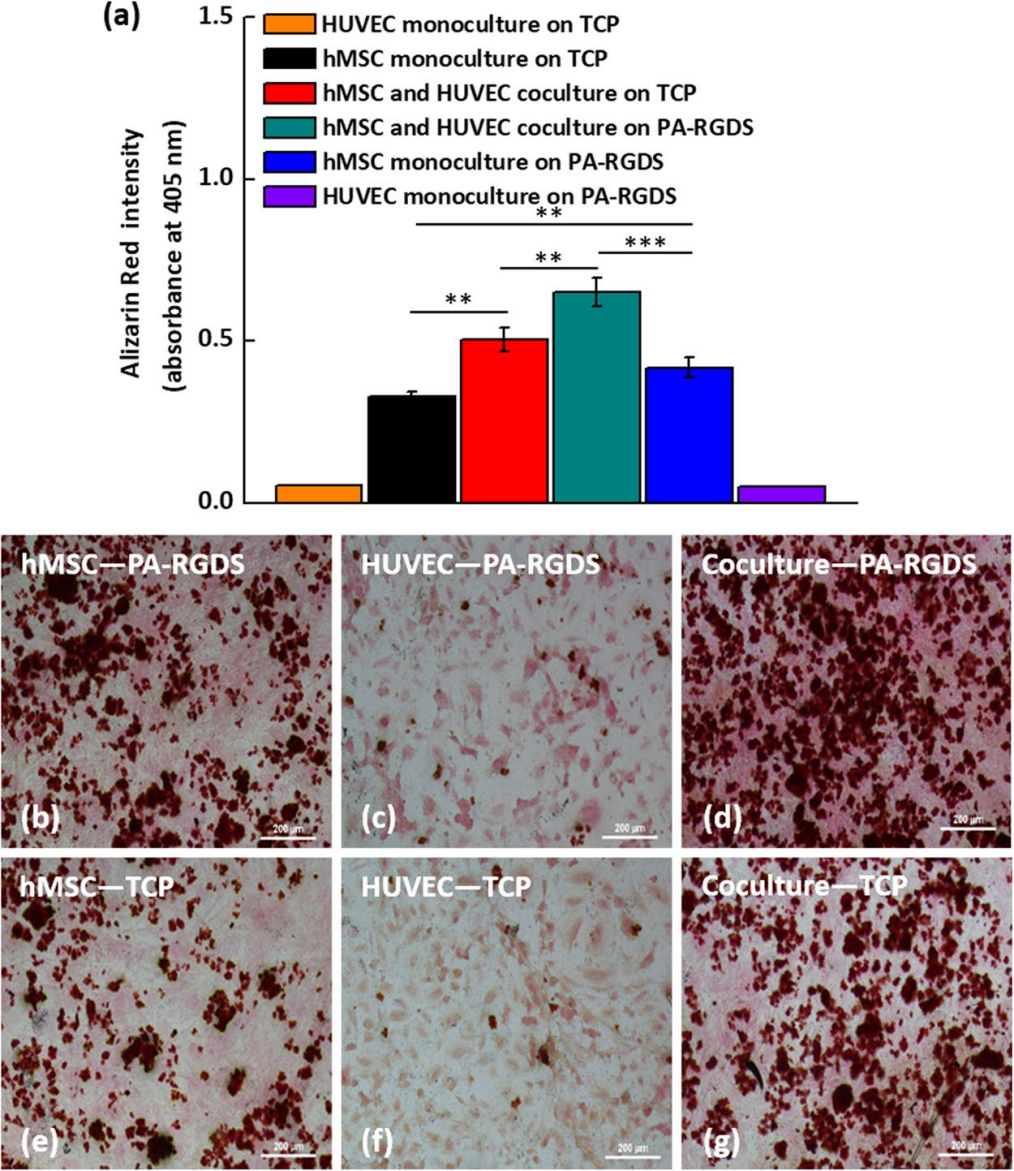
Day 21 Alizarin Red Quantification (**a**). Day 28 von Kossa staining. Row 1: PA-RGDS (**b**–**d**), and Row 2: TCP (**e**–**g**). Column 1: hMSCs (**b**,**e**), Column 2: HUVECs (**c**,**f**), Column 3: Coculture (**d**,**g**). Red staining shows locations of calcium deposits.

When osteogenic cells start to differentiate into osteoblasts, they begin to secrete mineral deposits^35^. Therefore, high mineral accumulation in the ECM usually indicates complete osteogenic differentiation of osteoblasts. In addition, ALP activity has a significant role in initiating mineralization. Since the synergistic promotion of osteogenic differentiation of hMSCs by combining coculture and PA-RGDS was observed earlier, we next investigated the mineralization degree in the six groups of interest after a 21-day culture period using the Alizarin Red quantification kit. The Alizarin Red quantification for calcium deposition shows that mineralization induced by coculture and PA-RGDS was significantly higher than that of the other studied groups (Fig. 3a). The intensity of the absorbance of Alizarin Red in the coculture on PA-RGDS, coculture on TCP, monoculture on TCP and monoculture on PA-RGDS are 0.65 ± 0.04, 0.50 ± 0.03, 0.32 ± 0.014, and 0.42 ± 0.03, respectively (Fig. 3a). A comparable result was also observed in Alizarin Red quantification after 28-day culture: the mineralization resulting from the coculture and PA-RGDS achieved the highest calcium deposits among all the studied groups (see Supplementary Fig. S1).

Moreover, to better visualize the mineralization degree, we characterized the extent of mineralization in the groups after a 28-day culture period using von Kossa staining. The reason we chose von Kossa staining is that calcium deposits stained by von Kossa staining are easier to observe than those stained by Alizarin red staining. As seen in Fig. 3b–g, the von Kossa staining results agree well with the result observed in Alizarin Red quantification. Specifically, von Kossa staining for calcium shows that cocultures contained a larger mineralized area with more mineral deposition than monocultures. In addition, hMSCs on PA-RGDS displayed greater mineralization than those on TCPs. This observation indicates the improved osteoconductive and osteoinductive potential of PA-RGDS cultures due to cell interactions with the RGDS ligand sequence (found in numerous ECM proteins), which may have triggered specific differentiation cues via cell-ECM interactions^29,40,41^. Moreover, the RGDS cell adhesion moiety has been shown to enhance osteoblast cell attachment and spreading, as well as induce mineralization^42–44^. Notably, cocultures on PA-RGDS exhibited the greatest degree of mineralization, as revealed by the larger stained area of mineral deposits. This result is consistent with the data collected from the ALP assay and further supports that osteogenesis is significantly enhanced when hMSCs are cocultured with HUVECs on PA-RGDS. Furthermore, Grellier *et al*. observed a similar result in an earlier study in which mineralization was more extensive when osteoprogenitors were cocultured with endothelial cells, rather than monocultured, and when both cells were immobilized on RGD-grafted alginate microspheres^45,46^. As previously mentioned, the peak ALP activity in cocultured hMSCs on PA-RGDS was observed at day 14, signifying that the differentiation process had begun prior to this day. During cell differentiation, peak ALP activity occurs before mineralized matrix formation, as ALP promotes mineralization^34,35^. Therefore, we speculate that mineralization started in PA-RGDS cocultures before other cultures. As shown in von Kossa staining images and Alizarin red quantification, accelerated initiation of the mineralization process in the PA-RGDS cocultures resulted in the greatest amount of mineral accumulation on both day 21 and day 28.

### Osteogenic and angiogenic gene expression and protein secretion

In addition to ALP activity and mineralization, the gene expressions of several crucial osteogenic markers were quantified for all samples at 7, 14, and 21 days post cell-seeding. Analyzed osteogenic gene markers included runt-related transcription factor 2 (Runx2), bone morphogenic protein-2 (BMP-2), ALP, and osteocalcin (OCN). During the well-orchestrated process of hMSC differentiation into osteoblasts, these genes, which are indicative of osteogenic phenotypes, are upregulated in a specific sequence. Thus, they are considered established markers of osteoblastic development^47^.

**Figure 4:**
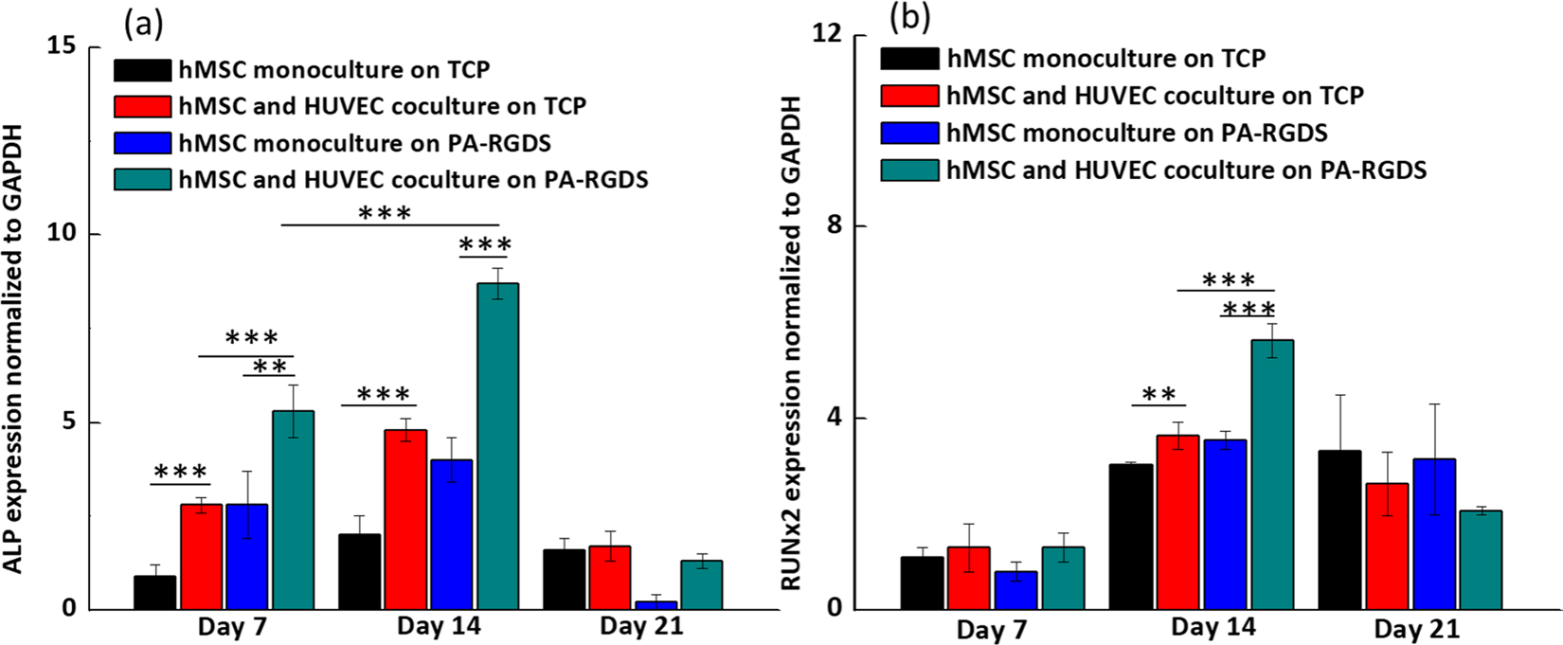
ALP (**a**) and Runx2 (**b**) gene expression. Gene expression is shown for monocultures and cocultures on TCPs and PA-RGDS at days 7, 14, and 21, as expressed as fold ratios relative to gene expression of hMSC monocultures on TCPs at day 7. Data, provided as mean ± standard deviation, are normalized to GAPDH gene expression (**p < 0.05 and ***p < 0.005).

ALP gene expression was investigated first. As seen in Fig. 4a, on days 7 and 14, ALP gene expression was significantly upregulated in PA-RGDS cocultures as compared to other cultures. Upregulation of ALP in PA-RGDS cocultures helps explain the enhanced mineralization and ALP content in these cultures. As an early marker for osteogenesis, ALP gene expression peaked on day 14 in the PA-RGDS cocultures at a higher value than in all other cultures which signifies the heightened osteogenic potential of this test group. Though, on days 7 and 14, hMSC monocultures on PA-RGDS showed enhanced ALP gene expression compared to monocultures on TCPs gene expression was not as pronounced as that seen in PA-RGDS cocultures. In previous studies, endothelial cell and hMSC cocultures showed similar early upregulation of ALP expression compared to hMSC monocultures^16,36,48,49^. However, as seen in this study, PA-RGDS promotes even higher levels of early ALP gene expression. Moreover, a downregulation of ALP gene expression was observed on day 21 in cocultures on PA-RGDS nanomatrix, which further suggests that PA-RGDS nanomatrix combined with coculture can accelerate the osteogenic differentiation of hMSCs in coculture. This downregulation might be attributed to the development of an osteoid matrix around osteoblasts as mineralization progressed^47^.

Secondly, Runx2 gene expression was analyzed. Runx2 is the earliest transcriptional regulator necessary for bone formation and is also an essential mediator of osteoblast differentiation^50,51^. Seen in Fig. 4b, significant differences in Runx2 gene expression were not observed between any of the culture groups on day 7. A similar result was reported by Xue *et al*., in which coculturing hMSCs with HUVECs did not significantly increase their expression of Runx2 after 5 days of incubation compared with Runx2 gene expression in hMSC monocultures^16^. However, on day 14, coculturing substantially enhanced Runx2 gene expression as compared to that in hMSC monocultures. Similarly, Bidarra *et al*. also showed that Runx2 gene expression is upregulated in cocultures compared to monocultures on TCP^36^. Importantly, Runx2 gene expression in PA-RGDS cocultures was upregulated at day 14 more than all other cultures at that timepoint. This upregulation indicates that the RGDS cell adhesion ligand, coupled with the coculturing condition, can promote and accelerate Runx2 gene expression, leading to earlier phenotypic commitment of hMSCs to osteogenic differentiation. In addition, a decrease of Runx2 gene expression in PA-RGDS cocultures was seen on day 21. The interesting observation may be ascribed to the function and osteogenic cell source of Runx2; it is a transcription factor that increases the number of immature osteoblasts, which is strongly expressed by preosteoblasts and immature osteoblasts but downregulated by mature osteoblasts^52,53^. Thus, the observed upregulated expression of Runx2 on day 14 in cocultures on PA-RGDS indicates that a high number of hMSCs had differentiated into preosteoblasts and immature osteoblasts, but the subsequent downregulation supports the conclusion that most of these osteogenic cells had fully matured into osteoblasts by day 21.

**Figure 5:**
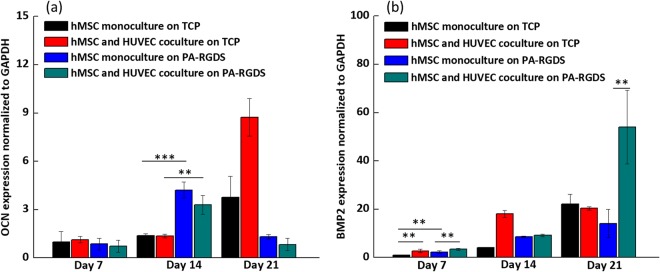
OCN (**a**) and BMP-2 (**b**) gene expression. Gene expression is shown for monocultures and cocultures on TCPs and PA-RGDS at days 7, 14, and 21 post cell-seeding, as expressed as fold ratios relative to gene expression of hMSC monocultures on TCPs at day 7. Data are normalized to GAPDH gene expression (**p < 0.05 and ***p < 0.005).

Runx2 regulates the expression of bone matrix protein genes—including OCN—during osteoblast differentiation^52^. Since an upregulation of Runx2 in PA-RGDS cocultures was observed, next we investigated OCN gene expression, which is regarded as an established late marker for osteoblast differentiation that appears concomitantly with the mineralization phase of bone formation^47^. As seen in Fig. 5a, the OCN gene expression at day 14 for monocultures and cocultures on PA-RGDS was almost 3.5 times higher than its counterparts on TCPs, indicating that PA-RGDS can promote OCN gene expression However, on day 14, we did not see a synergistic effect on gene expression produced by the combination of coculturing and PA-RGDS. Moreover, on day 21, significant downregulations of OCN gene expression were observed in monocultures and cocultures on PA-RGDS; conversely, an upregulation in OCN gene expression was observed in TCP monocultures and cocultures on day 21, indicating a prolonged delay in osteoinduction compared to that of cultures on PA-RGDS. Thus, PA-RGDS appears to be the most potent factor that fosters accelerated OCN expression. However, on day 21, coculturing upregulated gene expression on TCPs compared to monoculturing. As OCN is highly expressed by mature osteoblasts, the accelerated upregulation of OCN gene expression observed on PA-RGDS cultures at day 14 suggests that more hMSCs had terminally differentiated by this timepoint than those on TCPs^47,52^. The subsequent downregulation of gene expression observed in the PA-RGDS cultures at day 21 may have been a result of mature osteoblasts becoming osteocytes and embedding in the ECM they secreted^47,52^. Compared to days 7 and 14, amplified OCN gene expression in TCP cultures at day 21 may be indicative of an increased number of mature osteoblasts, which had not yet become embedded osteocytes.

BMP-2 gene expression is shown in Fig. 5b. Compared with hMSC monocultures on TCPs, an earlier upregulation of BMP-2 was seen in response to the coculture condition and the PA-RGDS substrate. On day 7, the combination of coculture and PA-RGDS amplified the expression of the BMP-2 gene more than the PA substrate alone. However, at day 14, BMP-2 gene expression was the most pronounced in cocultures on TCPs. By day 21, cocultures on PA-RGDS displayed the most enhanced BMP-2 gene expression, which was, on average, more than 50 times higher than hMSC monocultures on TCPs at day 7 As one of the most readily detectable BMPs in osteogenic cultures, BMP-2 promotes the differentiation of mesenchymal progenitor cells into osteoblasts^47^. Moreover, BMP-2 is the most powerful inducer of bone formation *in vivo*. Studies have demonstrated that high doses of BMP-2 will initiate earlier bone formation in osteogenic cultures^54^. BMP-2 gene expression is upregulated early in the process of bone development and, as a signaling molecule, facilitates the expression of Runx2 and ALP in osteoprogenitor cells^47,55^. Therefore, the earlier upregulation at day 7 of BMP-2 gene expression in cocultures on PA-RGDS may signify an accelerated initiation of hMSC osteogenic differentiation in contrast with other cultures. The second upregulation of BMP-2 at day 21 suggests that there was higher potential for bone formation in cocultures on PA-RGDS than in other test groups.

**Figure 6:**
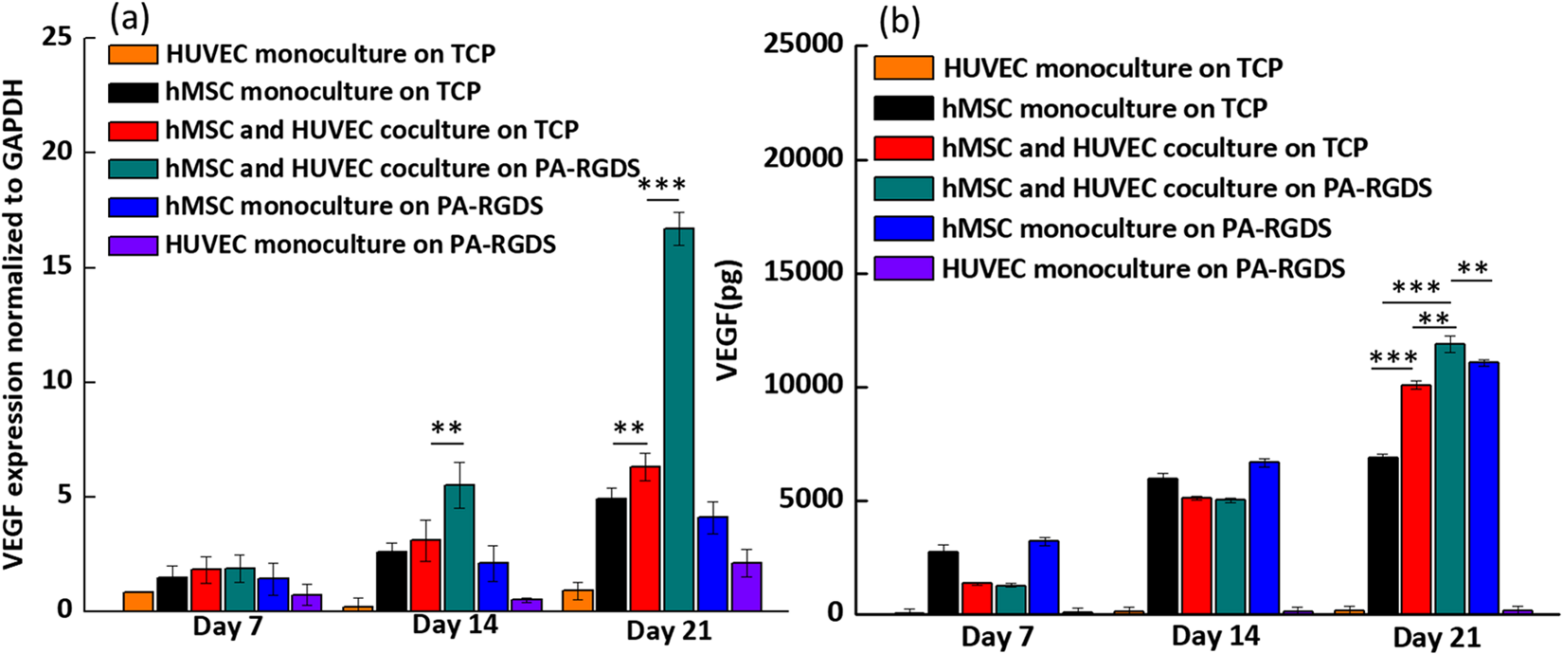
VEGF gene expression (**a**) and accumulative VEGF secretion (**b**). Gene expression is displayed for cultures on days 7, 14, and 21 as a fold ratio relative to HUVEC monocultures on TCP at day 7. Data are expressed as mean ± standard deviation and are normalized to GAPDH gene expression (**p < 0.05 and ***p < 0.005).

The expression of vascular endothelial growth factor (VEGF) correlates with osteoblastic differentiation. VEGF gene expression is low when osteoblastogenesis begins, it increases in parallel with OCN expression during terminal differentiation, and it peaks during mineralization^56^. Thus, VEGF is an osteogenic marker. As shown in Fig. 6a, on day 14 and day 21, cocultures on PA-RGDS exhibited substantially greater expression than all other culture conditions. On day 21, VEGF gene expression in cocultures on PA-RGDS was almost 10 times higher than that in TCP cocultures, and it was almost 15 and 12 times higher than HUVEC and hMSC monocultures on PA-RGDS, respectively. In addition, on day 21, cocultures on TCPs had slightly higher VEGF gene expression than hMSC monocultures on TCPs, which agrees with a former study that demonstrated that coculturing osteoprogenitor cells with HUVECs can result in higher VEGF gene expression than that seen in monocultures^57^. Taken together, these data indicate that the coculture condition and PA-RGDS substrate can significantly promote the osteogenesis of hMSCs^46^. The enhancement of VEGF gene expression observed here may also explain the elevated level of ALP activity in the PA-RGDS cocultures on day 14, as observed using the ALP assay^58^.

We also investigated the degree of VEGF secretion using enzyme-linked immunosorbent assay. Significantly, on day 21, the synergistic enhancement of VEGF secretion resulting from PA-RGDS and coculture was prominent, as demonstrated by the result that the secreted VEGF amount in the coculture on PA-RGDS was the highest (Fig. 6b). The greatest VEGF production at day 21 in coculture on PA-RGDS compared to other groups may be ascribed to the greatest gene upregulation of VEGF in this group at day 14 an day 21 (Fig. 6a). More importantly, VEGF secreted by osteoblasts/osteocytes has high angiogenic power, as it serves as a paracrine factor that promotes endothelial cell proliferation. Indeed, a previous study reported that VEGF-secreting osteoblastic cells, derived from hMSCs, increased the proliferation of HUVECs^59^. Thus, VEGF is also used as an angiogenic marker. Therefore, the augmented VEGF gene expression and protein secretion in PA-RGDS cocultures at day 21 indicate that the hMSCs reached an elevated level of osteogenic differentiation and were expected to stimulate more angiogenesis in this culture group than in any other test groups.

### Network formation imaging

**Figure 7:**
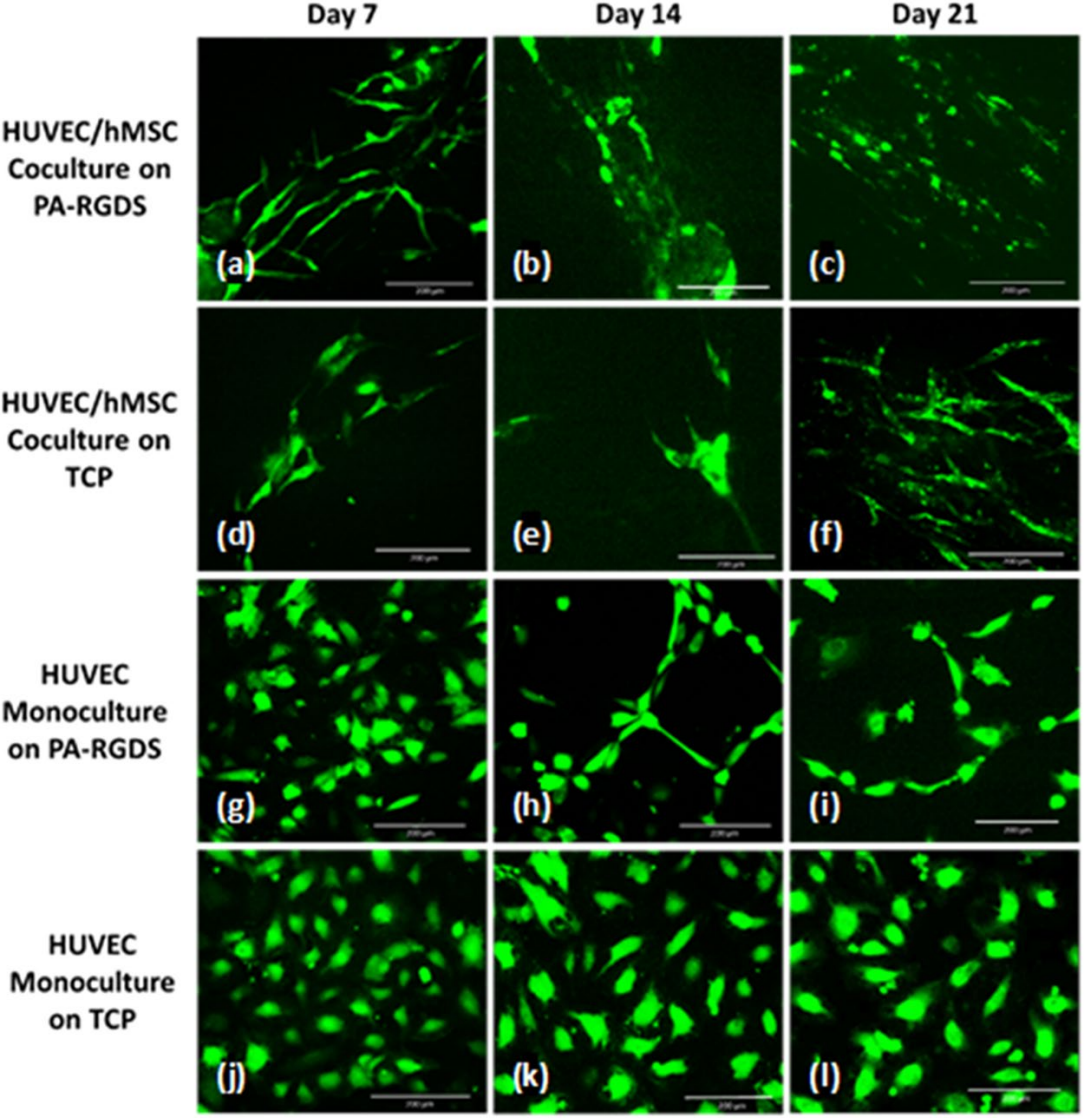
GFP-expressing HUVECs, indicating extent of network formation. Images are shown for HUVEC/hMSC cocultures on PA-RGDS (**a**–**c**) and on TCPs (**d**–**f**) and for HUVEC monocultures on PA-RGDS (**g**–**i**) and on TCPs (**j**–**l**). Fluorescent images show progression of vascular morphogenesis at days 7 (**a**,**d**,**g**,**j**), 14 (**b**,**e**,**h**,**k**), and 21 (**c**,**f**,**i**,**l**) post cell-seeding.

To check our hypothesis that increased VEGF gene expression and protein secretion would increase angiogenesis, we imaged the morphology of HUVECs and the extent of HUVEC network formation in each culture group. By day 7 post cell-seeding, HUVEC/hMSC cocultures on PA-RGDS showed aggregation and alignment of endothelial cells, as well as extensive pseudopodia formation characteristic of the vascular morphogenetic process (Fig. 7a). Cocultures on TCP did not display the same degree of extending pseudopodia as that seen in cocultures on PA-RGDS. By day 14, network formation, as indicated by further aggregation, spreading, and branching, was quite apparent in PA-RGDS cocultures (Fig. 7b). Cell elongation progressed slightly by day 21 in these cocultures (Fig. 7c). At day 14, cocultures on TCP showed considerate cell spreading but much less coalescence than cocultures on PA-RGDS (Fig. 7e). However, by day 21, the cell aggregation in TCP cocultures was much more reminiscent of that seen in PA-RGDS cocultures (Fig. 7f). In contrast to the cocultures, HUVEC monocultures showed much less vascular network formation. Nevertheless, PA-RGDS monocultures outperformed monocultures on TCPs. For instance, at day 14, HUVEC monocultures on PA-RGDS displayed some pseudopodia formation and cell clustering. However, from day 14 to day 21, vascular morphogenesis did not seem to progress. HUVECs in monocultures on TCPs showed little to no clustering and cell spreading throughout the duration of the study but remained relatively round and separated.

It has previously been reported by Issaragrisil’s group that the cytokines insulin-like growth factor 1, stromal cell-derived factor 1, and VEGF—all of which are secreted by hMSCs—can promote HUVEC vessel formation^60^. Thus, the accelerated endothelial cell spreading, aggregation, alignment and network formation in cocultures on PA-RGDS indicates that cell-cell interactions between osteogenic and endothelial cells are vital to promote vascular network formation, which may be further enhanced through interactions with the cell adhesion ligand RGDS provided by the functionalized PA.

In conclusion, we used ALP assay, von Kossa staining, Alizarin Red staining, analysis of osteogenic and angiogenic gene expression and related protein content, and characterization of endothelial cell network formation to evaluate the synergistic effects of PA-RGDS nanomatrix and coculturing hMSCs with HUVECs on hMSC osteogenesis and HUVEC angiogenesis. As compared to monocultures and all cultures on TCPs, the PA-RGDS nanomatrix and coculture condition synergistically enhanced ALP activity, mineralization character, and most of the studied osteogenic gene expression by hMSCs. Moreover, upregulated VEGF gene expression and increased VEGF secretion as well as network-like structures were observed in cocultures on PA-RGDS, hinting at a synergy between hMSCs and HUVECs and PA-RGDS that improved angiogenesis. The results of the current study clearly suggest that the PA-RGDS nanomatrix, combined with the beneficial cell-cell crosstalk in HUVEC/hMSC cocultures, offers a promising biomimetic solution for bone tissue engineering that provides an angiogenic and osteogenic environment with the potential to stimulate, and even accelerate, bone healing and growth.

## Methods

### PA-RGDS synthesis

Similar to previously described methods of peptide synthesis, PA-RGDS was synthesized in an Advanced Chemtech Apex 396 peptide synthesizer at a 0.30 mmol scale using standard Fmoc-chemistry^28,61^. The PA was alkylated by reacting N-termini of the peptides with 2 equivalents of palmitic acid, 2 equivalents of o-benzotriazole-N,N,N′,N′ tetramethyluroniumhexafluorophosphate, and 4 equivalents of diisopropylethylamine in dimethylformamide for 3 hours at room temperature. After repeating the alkylation reaction once more, cleavageand deprotection of PA-RGDS were performed through gentle mixing in a solution of trifluoroacetic acid (TFA), deionized (DI) water, and triisopropylsilane at the ratio of 38:1:1 for 1 hour at room temperature. The resulting solution was filtered from the resin, which was rinsed with 2 additional milliliters of TFA. The collected flow-through was rotoevaporated for further removal of impurities, and the PA was precipitated out of solution using cold ether. The precipitate was lyophilized for 48–72 hours.

### PA-RGDS nanomatrix

A 0.1 wt% stock solution of PA-RGDS was prepared in DI water and adjusted to pH 7.4 through the controlled addition of NaOH. For the alkaline phosphatase assay and von Kossa staining experiments, 200 uL of PA-RGDS stock solution were then placed in each well of 48-well TCPs. For the PCR experiments, 1 mL of PA-RGDS stock solution was placed in each well of 6-well TCPs. For Alizarin Red quantification as well as VEGF secretion study, 400 ul of PA-RGDS stock solution was placed in each well of 24-well TCPs. To evaporate the solvent and induce PA-RGDS self-assembly, the plates were placed in a biosafety cabinet for 24 hours and UV-sterilized for an additional 1.5 hours.

### Cell culture

hMSCs (Lonza, Walkersville, MD), isolated from bone marrow, were cultured in Mesenchymal Stem Cell Growth Medium. GFP-HUVECs (Angioproteomie, Boston, MA) were cultured in Endothelial Basal Medium (EBM) supplemented with the Endothelial Growth Medium-2 (EGM-2) SingleQuots kit. Each of these cell types were cultured in T-75 flasks (Corning Inc., Corning, NY) at standard culture conditions of 37 °C, 95% humidity, and 5% carbon dioxide. When confluent, the cells were passaged using 0.05% trypsin and then seeded in TCPs. The seeded cells were cultured at the previously described standard conditions. During both cell expansion and plate culture, media was replaced every 2 to 3 days.

For all experiments, the following conditions were used: PA-RGDS nanomatrix substrates, used for experimental groups, and TCPs, used for control groups. On each substrate, 3 cell-seeding conditions were used: 1) hMSCs in monoculture, 2) GFP-HUVECs in monoculture, and 3) hMSCs cocultured with GFP-HUVECs. In monocultures used for the alkaline phosphatase assay and von Kossa staining, 13,500 GFP-HUVECs or hMSCs were seeded per well. In cocultures, 13,500 hMSCs and HUVECs were each seeded per well, yielding a combined total of 27,000 cells per well of the 48-well plates. For Alizarin Red quantification as well as VEGF secretion study, 25,000 GFP-HUVECs or hMSCs were seeded per well. In cocultures, 25,000 hMSCs and HUVECs were each seeded per well, yielding a combined total of 50,000 cells per well of the 24-well plates. Six repetitions were used for each condition.

In the GFP-HUVEC and hMSC monocultures used for qRT-PCR experiments and network formation imaging, 80,000 cells were seeded per well. For cocultures, 80,000 hMSCs and GFP-HUVECs were each seeded per well, equaling a total of 160,000 cells per well of the 6-well plates. Each condition was repeated in quadruplicate. After cell seeding, a 1:1 volume ratio of osteogenic differentiation medium and endothelial cell growth medium was added to each well for cell culture. The osteogenic differentiation medium was made of a basal medium and an hMSC Osteogenic SingleQuots kit (Lonza, Walkersville, MD) that contained the following supplements: 0.5% dexamethasone, 0.5% ascorbate, 10% meningeal cell growth supplement, 2% L-glutamine, 1% penicillin-streptomycin, and 1% B-glycerophosphate. The endothelial cell growth medium consisted of EBM supplemented with the EGM-2 SingleQuots kit, which contained the following supplements: 2% fetal bovine serum, 0.04% hydrocortisone, 0.4% human fibroblast growth factor-B, 0.1% VEGF, 0.1% R3 insulin like growth factor-1, 0.1% ascorbic acid, 0.1% human epidermal growth factor, 0.1% GA-1000 (gentamicin, amphotericin-B), and 0.1% heparin (Lonza, Walkersville, MD).

### Alkaline phosphatase assay

Cells were harvested with trypsin at 1, 7, 14, and 28 days and stored in Eppendorf tubes at −80 °C until they were retrieved for analysis. An ALP fluorometric assay (Abcam, Cambridge, MA) was used to measure the amount of ALP from each sample. Specifically, 60 μL of cell lysate, 60 μL of alkaline buffer, and 100 μL of phosphatase substrate solution were added to each well of a 96-well plate and incubated for 1 hour at 37 °C. Standards in known concentrations ranging from 0 μM to 1,000 μM were prepared using p-nitrophenol and added to designated wells in the same plate. After incubating for 1 hour, the kinase reaction was stopped by adding 100 μL of 0.3 M NaOH to each well. Absorbances were measured at 405 nm using a microplate reader (Synergy HT, BIO-TEK Instruments, Winooski, VT) and then compared to standards with known ALP content. Results were normalized to the total cell number at each timepoint, as measured by the PicoGreen DNA Assay (Molecular Probes, Eugene, OR). More precisely, Picogreen Dye from the assay kit was added to prepared samples, which were incubated in the dark for 15 minutes. The amount of DNA was then measured using a fluorescence microplate reader and was compared to prepared standards with known DNA content. Finally, ALP results were normalized to measured DNA content.

### Von Kossa staining

After 28 days, cells were fixed with 10% formalin according to the manufacturer’s protocol (Abcam, San Francisco, CA). Stained cells were imaged using color brightfield microscopy.

### Alizarin Red staining and quantification

After 21 and 28 day culture, Alizarin Red quantification was conducted through the Alizarin Red Quantification Assay **(**ScienCell, Carlsbad, CA**)** according to the manufacturer’s protocol using a microplate reader (Synergy HT, BIO-TEK Instruments, Winooski, VT).The absorbance was measured at 405 nm.

### qRT-PCR

At days 7, 14, and 21, GFP-HUVECs and hMSCs were collected and lysed using RNeasy Plus Mini Kit (Qiagen, Hilden, Germany). RNA from the cells was isolated according to the manufacturer’s protocol. RNA was suspended in nuclease free water, and an ND-1000 UV spectrophotometer (Nanodrop, Wilmington, DE) was used to quantify the concentration of RNA for each sample. Complementary DNA was then synthesized using 500 ng of RNA, which was reverse transcribed in a 2720 Thermo Cycler (Applied Biosystems, Foster City, CA) using a Verso cDNA Synthesis Kit (Thermo Fisher Scientific, Waltham, MA) according to the manufacturer’s protocol. Samples were prepared in a 96-well PCR plate using the TaqMan Master Mix protocol. Each sample consisted of 2 µL of cDNA solution, 10 µL of 2x master mix, 7 µL of RNA-free water, and 1 µL of gene primer (Runx2, BMP-2, ALP, OCN, VEGF, or GAPDH gene primer) from a TaqMan Gene Expression Assay kit (Applied Biosytems, Foster City, CA). The PCR plate was run in a LightCycler 480 (Roche Life Science, Indianapolis, IN) for the following cycles: pre-incubation at 50 °C for 2 minutes and 95 °C for 10 min; amplification for 45 cycles, at 95 °C for 15 seconds and 60 °C for 1 minute during each cycle; melting at 95°C for 5 seconds and 65 °C for 1 minute; and cooling at 40^o^C for 30 seconds. Runx2, BMP-2, ALP, and OCN gene amplifications were used to evaluate osteogenic gene expression. VEGF pathway gene amplification was used to evaluate angiogenic gene expression. Gene expression was normalized against the housekeeping gene, GAPDH. As described in a previous study, the 2^−ΔΔCT^ method was used to assess gene expression^29^. For Runx2, BMP-2, ALP, and OCN gene expression, data at each timepoint is expressed as a fold ratio relative to data acquired for the hMSC monoculture on TCP at day 7. VEGF gene expression at each timepoint is shown as a fold ratio relative to the GFP-HUVEC monoculture on TCP at day 7.

### Evaluation of VEGF secretion

To measure the amount of secreted VEGF from the six groups of interests, enzyme-linked immunosorbent assay (ELISA; R&D system, MN, USA) was conducted. Supernatant from the samples was collected every other day, and the accumulative secretion of VEGF on day 7, 14 and 21 were analyzed by sandwich ELISA method using a microplate reader (Synergy HT, BIO-TEK Instruments, Winooski, VT). The absorbance was measured at 450 nm.

### Network formation imaging

Using fluorescence microscopy, GFP-HUVECs were imaged at 7, 14, and 21 days post cell-seeding to assess the extent of cell spreading, alignment, and aggregation.

### Statistical analyses

Presented data are representative of experimental results. Each experiment was performed at least three independent times with conditions repeated in quadruplicate at each timepoint. Values are expressed as mean ± standard deviation. To assess significance between data, Student’s t test or one-way analysis of variance (ANOVA) and post hoc analysis using Tukey’s range test for multiple comparisons were conducted. For all tests, *p* < 0.05 is considered statistically significant. *p* < 0.005 is considered extremely statistically significant. Moreover, the *p* values for Fig. 3a are summarized in Supplementary Table S1. The *p* values for Figs 4–5 are summarized in Supplementary Table S2. The *p* values for Fig. 6 are summarized in Supplementary Table S3.

## Electronic supplementary material

Supplementary Information

## Data Availability

The datasets generated during the current study are available from the corresponding author on reasonable request.
