# Supplementary material for: Angiogenic and Osteogenic Synergy of Human Mesenchymal Stem Cells and Human Umbilical Vein Endothelial Cells Cocultured on a Nanomatrix
Source: Sci Rep. 2018 Oct 24;8:15749. doi: 10.1038/s41598-018-34033-2 (PMC6200728; doi:10.1038/s41598-018-34033-2)
Supplement: Supplementary file 1 — Supplementary Information [file 41598_2018_34033_MOESM1_ESM.docx]

**Supplementary Information**

**Angiogenic and Osteogenic Synergy of Human Mesenchymal Stem Cells and Human Umbilical Vein Endothelial Cells Cocultured on a Nanomatrix**

Jun Chen^1+^, Lily Deng^1+^, Catherine Porter^1^, Grant Alexander^1^, Dhruv Patel^1^, Jeremy Vines^1^, Xixi Zhang^1^ David Chasteen-Boyd^1^*,* Hak-Joon Sung^2^, Yi-Ping Li^3^, Amjad Javed^4^, Shawn Gilbert^5^, Kyounga Cheon^6^, and Ho-Wook Jun^1^*


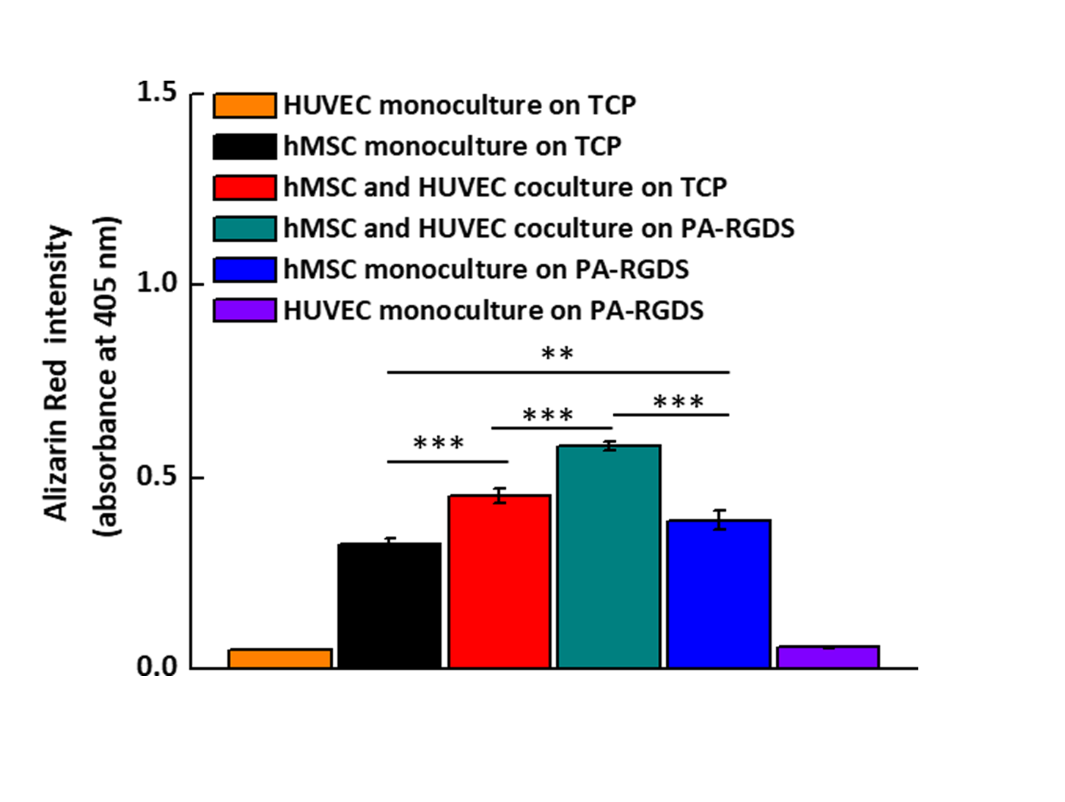


**Figure.S1** Day 28 Alizarin Red Quantification

**Supplementary Table S1**. *p* value summary of Alizarin Red data shown in Figure 3a and Figure S1. p < 0.05 is considered statistically significant. p < 0.005 is considered extremely significant.

| **Day 21** | ***p* values** |
| --- | --- |
| Coculture-PA-RGDS vs Coculture-TCP | 0.0104 |
| Coculture-PA-RGDS vs hMSC-PA-RGDS | 0.0017 |
| hMSC-TCP vs Coculture-TCP | 0.0014 |
| hMSC-TCP vs hMSC-PA-RGDS | 0.0118 |
| **Day 28** | ***p* values** |
| Coculture-PA-RGDS vs Coculture-TCP | 0.0007 |
| Coculture-PA-RGDS vs hMSC-PA-RGDS | 0.0002 |
| hMSC-TCP vs Coculture-TCP | 0.0009 |
| hMSC-TCP vs hMSC-PA-RGDS | 0.0181 |

**Supplementary Table S2.** *p* value summary of gene expression data shown in Figure 3-5. *p* < 0.05 is considered statistically significant. *p* < 0.005 is considered extremely significant.

| **ALP** | ***p* value** | **RUNx2** | ***p* value** |
| --- | --- | --- | --- |
| hMSC-TCP, 7D vs hMSC-PA-RGDS, 7D | 0.0256 | hMSC-TCP, 14D vs Coculture-TCP, 14D | 0.0061 |
| hMSC-TCP, 7D vs Coculture-PA-RGDS, 7D | 0.0006 | Coculture-PA-RGDS, 14D vs  Coculture-TCP, 14D | 0.0002 |
| hMSC-TCP, 7D vs Coculture-TCP, 7D | 0.0008 | Coculture-PA-RGDS, 14D vs  hMSC-PA-RGDS, 14D | 0.0004 |
| Coculture-PA-RGDS, 7D vs hMSC-PA-RGDS, 7D | 0.0191 | **OCN** | ***p* value** |
| Coculture-PA-RGDS, 7D vs Coculture-TCP, 7D | 0.0040 | Coculture-PA-RGDS, 14D vs Coculture-TCP, 14D | 0.0052 |
| Coculture-PA-RGDS, 14D vs Coculture-PA-RGDS, 7D | 0.0019 | hMSC-PA-RGDS, 14D vs hMSC-TCP, 14D | 0.0007 |
| Coculture-PA-RGDS, 14D vs Coculture-TCP, 14D | 0.0002 |  |  |
| **BMP-2** | ***p* value** |  |  |
| hMSC-TCP, 7D vs  Coculture-TCP, 7D | 0.0124 |  |  |
| Coculture-PA-RGDS, 7D vs hMSC-PA-RGDS, 7D | 0.0382 |  |  |
| hMSC-PA-RGDS, 7D vs hMSC-TCP, 7D | 0.0130 |  |  |
| Coculture-PA-RGDS, 21D vs hMSC-PA-RGDS, 21D | 0.0132 |  |  |
| Coculture-PA-RGDS, 21D vs  Coculture-TCP, 21D | 0.0189 |  |  |
| Coculture-PA-RGDS vs hMSC-TCP, 21D | 0.0259 |  |  |

**Supplementary Table S3.** *p* value summary of VEGF gene and protein expression data shown in Figure 6. *p* < 0.05 is considered statistically significant. *p* < 0.005 is considered extremely significant.

| **gene expression** | ***p* values** |
| --- | --- |
| Coculture-PA-RGDS,14D vs Coculture-TCP, 14D | 0.0135 |
| Coculture-PA-RGDS,21 D vs hMSC-TCP, 21D | 0.056 |
| Coculture-PA-RGDS,21D vs Coculture-TCP, 21D | 0.0001 |
| **protein expression** | ***p* values** |
| hMSC-TCP,21D vs hMSC-PA-RGDS,21D | 0.0118 |
| hMSC-PA-RGSD,21D vs Coculture-PA-RGDS,21D | 0.0017 |
| hMSC-TCP,21D vs Coculture-TCP, 21D | 0.0014 |
| Coculture-PA-RGDS,21D vs Coculture-TCP, 21D | 0.0104 |
